# 2231. Point Prevalence Survey on Antimicrobial Appropriateness in a Tertiary Care Hospital in Saudi Arabia

**DOI:** 10.1093/ofid/ofad500.1853

**Published:** 2023-11-27

**Authors:** Reem AlMahasnah, Hussain AlQassim, Ibtihal AlQaysum, Manal Atta, Faris AlRashidi, Hanan Abufara, Mai Hashhoush, Noura AlAjmi, Yamama AlJishi

**Affiliations:** King Fahad Specialist Hospital - Dammam, Dammam, Ash Sharqiyah, Saudi Arabia; King Fahad Specialist Hospital, Dammam, Ash Sharqiyah, Saudi Arabia; King Fahad Specialist Hospital, Dammam, Ash Sharqiyah, Saudi Arabia; King Fahad Specialist Hospital, Dammam, Ash Sharqiyah, Saudi Arabia; King Fahad Specialist Hospital, Dammam, Ash Sharqiyah, Saudi Arabia; King Fahad Specialist Hospital, Dammam, Ash Sharqiyah, Saudi Arabia; King Fahad Specialist Hospital, Dammam, Ash Sharqiyah, Saudi Arabia; King Fahad Specialist Hospital, Dammam, Ash Sharqiyah, Saudi Arabia; King Fahad Specialist Hospital, Dammam, Ash Sharqiyah, Saudi Arabia

## Abstract

**Background:**

Antimicrobial resistance is a significant global health problem. Optimizing the use of antimicrobial agents is a key strategic objective for any antimicrobial stewardship program (ASP). In the literature, 9-64% of antimicrobials prescriptions were inappropriate the hospital setting. We aim to evaluate antimicrobial utilization practices and subsequently implement informed interventions to optimize antimicrobial use by conducting a point prevalence survey (PPS).

**Methods:**

This is a single-center PPS at King Fahad Specialist Hospital-Dammam, Saudi Arabia. The hospital is specialized in Transplant, Oncology/hematology, and Neuroscience, with approximately 350 beds. All inpatient wards were conducted over one week period ward by ward. All inpatients on active antimicrobials were included. Data were collected from patients’ electronic medical records based on the WHO methodology for PPS on antibiotic use in hospitals. The outcome (appropriateness of antimicrobial treatment) included five elements: indication, duration, administration route, spectrum, and dosing. Assessments were made according to the local and international guidelines and the ASP team (Infectious diseases consultant and pharmacist).

**Results:**

A total of 316 patients’ medical records were screened. Of whom 121 were included. Nineteen (16%) patients were pediatrics, and 102 (84%) were adults. Patients’ characteristics are presented in **Table 1**. A total of 207 systemic antimicrobials were recorded, 117/207 (57%) therapeutic and 90/207 (43%) prophylactic. The antimicrobials were inappropriate in 73 /207 (35%) antimicrobial orders that included 84 elements. Eleven out of the 73 orders have more than one inappropriate element. The main reasons were inappropriate indication 31/84 (37%), followed by the antimicrobial spectrum 21/84 (25%). **Figure 1**

**Table 1:**
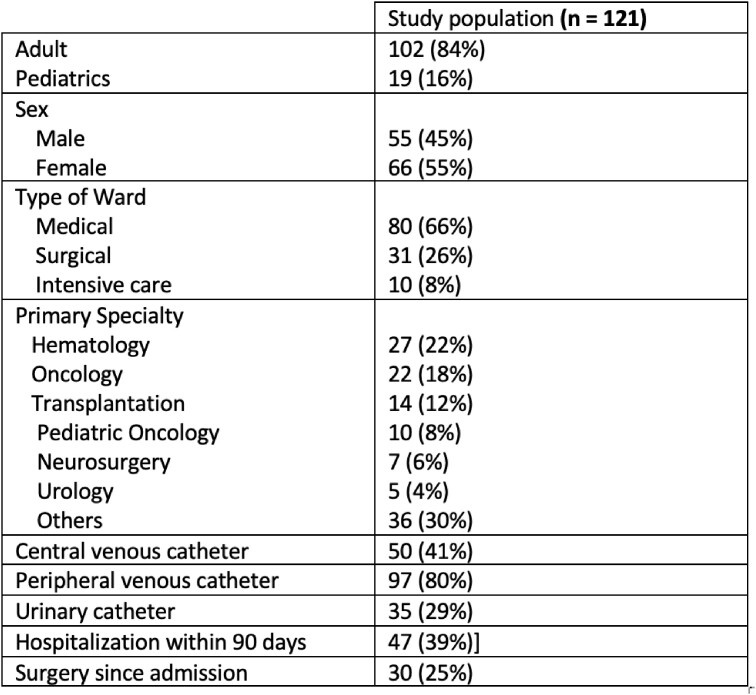
Characteristics of patients receiving systemic antimicrobial treatment, n (%)

**Figure 1:**
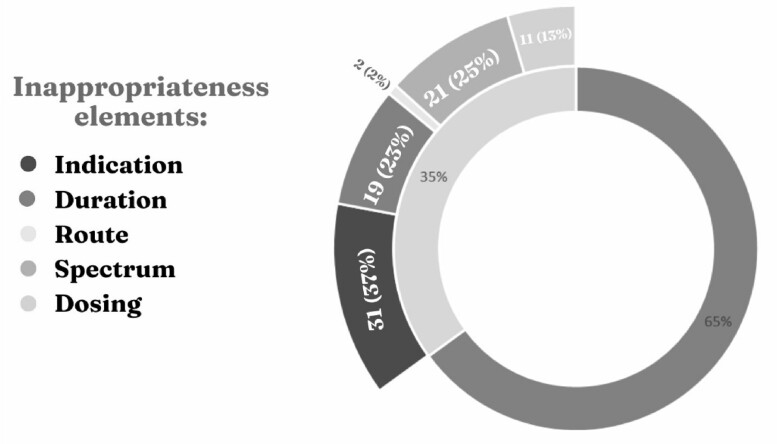
Elements of antimicrobials inappropriateness

**Conclusion:**

This point prevalence survey showed 35% of the antimicrobial prescription in the inpatient setting was inappropriate. The most common reasons were the indication followed by the spectrum and duration of antimicrobials. These findings provide valuable insight into stewardship's best approach and interventions to improve antimicrobials appropriateness.

**Disclosures:**

**All Authors**: No reported disclosures

